# In Vitro and In Vivo Preventive Effects of Thymoquinone against Breast Cancer: Role of DNMT1

**DOI:** 10.3390/molecules29020434

**Published:** 2024-01-16

**Authors:** Mohammed Kaleem, Asaad Kayali, Ryan A. Sheikh, Abudukadeer Kuerban, Mohammed A. Hassan, Naif Abdullah R. Almalki, Fahad A. Al-Abbasi, Firoz Anwar, Ziad Omran, Mahmoud Alhosin

**Affiliations:** 1 Department of Biochemistry, Faculty of Science, King Abdulaziz University, Jeddah 21589, Saudi Arabia; kaleemmubin88@gmail.com (M.K.); asaadkayali@hotmail.com (A.K.); rsheikh@kau.edu.sa (R.A.S.); abdukadir830@gmail.com (A.K.); maah3512@gmail.com (M.A.H.); naralmalki@kau.edu.sa (N.A.R.A.); alabassif@hotmail.com (F.A.A.-A.); firoz_anwar2000@yahoo.com (F.A.); 2 Department of Pharmacology, Dadasaheb Balpande College of Pharmacy, Rashtrasant Tukadoji Maharaj Nagpur University, Nagpur 440037, Maharashtra, India; 3 Department of Biomedical Sciences, College of Health Science, Abu Dhabi University, Abu Dhabi P.O. Box 59911, United Arab Emirates; 4 Experimental Biochemistry Unit, King Fahad Medical Research Centre, King Abdulaziz University, Jeddah 21589, Saudi Arabia; 5 Department of Pharmacy, College of Medicine and Health Sciences, Hadhramout University, Mukalla P.O. Box 8892, Yemen; 6 King Abdullah International Medical Research Center, King Saud Bin Abdelaziz University for Health Sciences, Jeddah 21423, Saudi Arabia; omranz@kaimrc.edu.sa; 7 King Abdulaziz Medical City, Ministry of National Guards-Health Affairs, Jeddah 21423, Saudi Arabia; 8 Cancer and Mutagenesis Unit, King Fahd Medical Research Center, King Abdulaziz University, Jeddah 21589, Saudi Arabia

**Keywords:** breast cancer, thymoquinone, metastasis, epigenetics, DNMT1

## Abstract

Breast cancer (BC) is one of the most common cancers in women and is a major cause of female cancer-related deaths. BC is a multifactorial disease caused by the dysregulation of many genes, raising the need to find novel drugs that function by targeting several signaling pathways. The antitumoral drug thymoquinone (TQ), found in black seed oil, has multitargeting properties against several signaling pathways. This study evaluated the inhibitory effects of TQ on the MCF7 and T47D human breast cancer cell lines and its antitumor activity against BC induced by a single oral dose (65 mg/kg) of 7,12-dimethylbenzanthracene (DMBA) in female rats. The therapeutic activity was evaluated in DMBA-treated rats who received oral TQ (50 mg/kg) three times weekly. TQ-treated MCF7 and T47D cells showed concentration-dependent inhibition of cell proliferation and induction of apoptosis. TQ also decreased the expression of DNA methyltransferase 1 (DNMT1) in both cancer cell types. In DMBA-treated animals, TQ inhibited the number of liver and kidney metastases. These effects were associated with a reduction in DNMT1 mRNA expression. These results indicate that TQ has protective effects against breast carcinogens through epigenetic mechanisms involving DNMT1 inhibition.

## 1. Introduction

Breast cancer (BC) is a complex disease characterized by distinct biological subtypes and numerous targeted prognostic markers of therapeutic importance [1,2]. In 2012, 1.7 million BC cases and 521,900 deaths were reported [3]. Over 2.3 million (11.7%) new cases and 684,996 (6.9%) mortalities from BC occurred in 2020, and its prevalence is expected to increase to over 3 million new cases and 1 million deaths by 2040, making BC the most common type of malignancy worldwide in women [4]. BC develops through several mechanisms, including epigenetic modifications that mainly involve the inactivation of tumor suppressor genes (TSGs) through DNA methylation [5,6,7]. Several studies have shown epigenetic inactivation of the TSG breast cancer susceptibility gene 1 (*BRCA1*) through promoter hypermethylation catalyzed by DNA methyltransferase 1 (DNMT1) [8,9,10]. DNMT1 overexpression is associated with BC development, and its downregulation has been reported to inhibit the proliferation and invasion of BC cells and the progression of BC [11,12,13]. Higher expression levels of DNMT1 have been found in patients with BC than in those with breast fibroadenoma [14]. DNMT1 overexpression has also been correlated with promoter hypermethylation, decreased expression of *BRCA1*, and lymph node metastasis [14]. These findings suggest that DNMT1 may be involved in the progression of breast cancer through an epigenetic inactivation of *BRCA1*. Abnormal DNA methylation is also associated with drug resistance in breast cancer cells [15,16], indicating that DNMT1 overexpression could also be a cause of BC resistance to therapy. Thus, one approach aimed at increasing the sensitivity of BC cells to chemotherapy is the inhibition of DNMT1 expression using the FDA-approved nucleoside DNMT inhibitors, azacytidine and decitabine. However, both drugs have adverse side effects and poor chemical stability [17,18]. The identification of novel, potent, and safe inhibitors of DNMT is therefore becoming increasingly important.

Plant-derived bioactive components may represent agents that could help overcome the challenge of finding safe DNMT inhibitors. For example, epigallocatechin-3-gallate (EGCG) [19,20] and curcumin [21,22] have anticancer activities related to their effects on DNA methylation and subsequent inhibition of the enzymatic activity and/or transcription of DNMT1. Another compound, thymoquinone (TQ), the biologically active component of black cumin (*Nigella sativa*) seeds, also shows cytotoxicity activities against various human cancer cells by targeting several signaling pathways, including the epigenetic machinery [23,24,25,26,27]. Several in vitro studies have shown that TQ has inhibitory effects on leukemia, a hematological tumor model, by inhibiting DNMT1 activity [28] and decreasing its expression at both the transcriptional and protein levels [25,27,29,30].

In the present study, we investigated the possibility that TQ would show similar inhibitory effects in vitro on the MCF7 and T47D human breast cancer cell lines and in vivo in a rat model of 7,12-dimethylbenzanthracene (DMBA)-induced BC through its ability to modulate DNMT1 expression.

## 2. Results

### 2.1. TQ Inhibited Growth and Induced Apoptosis in T47D and MCF7 Cells

**Figure 1:**
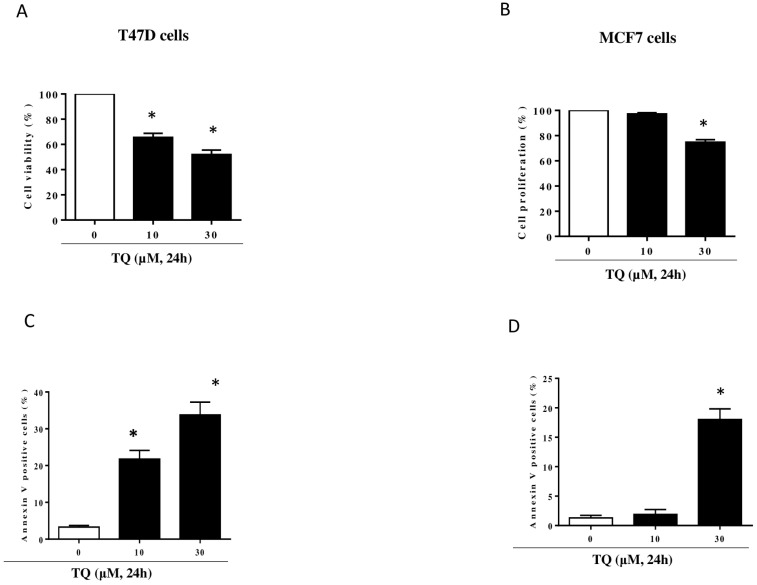
Effect of TQ on MCF7 and T47D cell proliferation and apoptosis. MCF7 cells (**A**,**C**) and T47D cells (**B**,**D**) were exposed to increasing concentrations of TQ at 10 or 30 µM for 24 h. Cell proliferation rate was assessed by WST-1 assay. Apoptosis was assessed by flow cytometry using the Annexin V/7AAD staining apoptosis. The data is representative of three different experiments. Values are shown as means ± S.E.M. (n = 3); *, *p* < 0.05 versus respective control.

The effect of TQ on T47D and MCF7 cell proliferation was examined by treating the cells with TQ for 24 h (Figure 1A,B). TQ induced half-maximum effects (IC50) on cell proliferation at concentrations of approximately 30 µM in T47D (Figure 1A) and 50 µM in MCF-7 in T47D cells, TQ concentration-dependently inhibited cell proliferation inhibition (Figure 1A) and triggered apoptosis (Figure 1C). The treatment of MCF7 cells with 30 μM TQ significantly decreased cell proliferation (Figure 1B) and increased the percentage of apoptotic cells (Figure 1D). These results show that TQ inhibits breast cancer cell proliferation through what was largely determined to be an apoptotic process.

### 2.2. TQ Reduced DMBA-Induced Kidney and Liver Damage

**Figure 2:**
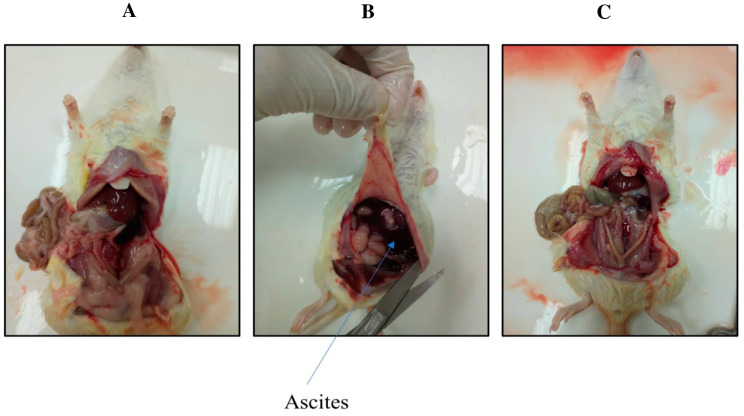
Morphology of the three groups of rats: the control group (**A**), the DMBA-induced rats (**B**), and the preventive group (**C**).

**Figure 3:**
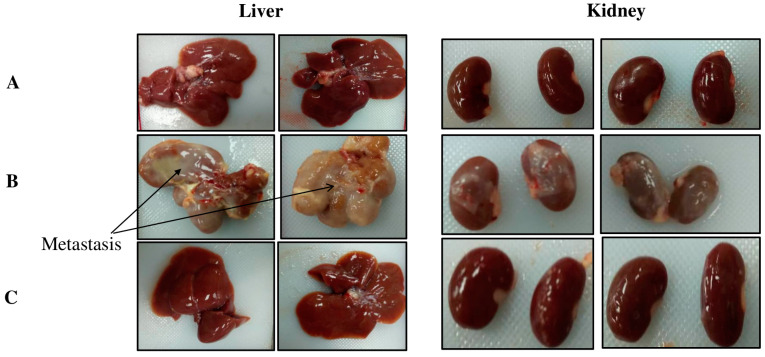
Liver and kidney morphology of the three groups of rats: the control group (**A**), the DMBA-induced rats (**B**), and the preventive group (**C**).

In group 1, the control group rats showed normal kidney and liver tissue morphology (Figure 2 and Figure 3A). In group 2, the DMBA-treated rats showed ascites and metastasis (Figure 2 and Figure 3B). The rats in group 3 (the preventive group) showed near-normal architecture, with reduced kidney and liver damage (Figure 2 and Figure 3C). The latter finding indicated that TQ had preventive effects on DMBA-induced kidney and liver damage, as these tissues in group 3 showed morphology nearly equivalent to that of the control group 1.

### 2.3. Effect of TQ on DMBA-Induced Histopathological Changes in Mammary Glands of Female Rats

**Figure 4:**
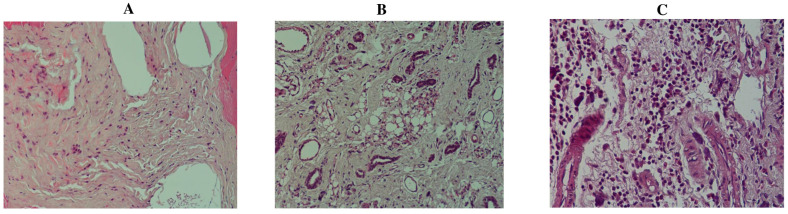
Effect of TQ on histopathology of mammary tissue from rats with DMBA-induced breast cancer. Data are shown as means ± S.E.M. (n = 12): rat mammary tissues from histological sections exhibit the typical anatomy of the control group’s mammary glands (×200) (**A**). (**B**) group 2: (DMBA (65 mg/kg, b. wt.) (×200)): mammary hypertrophy (×200) indicated an increase in the number of tiny ducts, and ductal hyperplasia and the sloughing of epithelial cells into the duct were among the neoplastic changes. (**C**) the preventive group (DMBA (65 mg/kg, b. wt.) and TQ (50 mg/kg, b. wt.) (×400)); displayed a decreased number of lobular alveolar damage and an increased number of acini.

Figure 4 depicts the typical mammary gland morphology as well as the pathological modifications caused by the administration of DMBA and TQ. The mammary glands of control group 1 showed normal tiny ducts with a single layer of epithelial cells surrounding them and a normal number of acini (Figure 4A). DMBA treatment of the rats induced the proliferation of the terminal ductal breast tissue and the formation of premalignant and malignant hyperplastic lesions that closely resemble human breast cancer. The morphological changes were characterized by an increased number of small ducts that were further differentiated into lobular units with striking secretion and neoplasia, including ductal hyperplasia and sloughing of epithelial cells into the duct. No edema, inflammation by neutrophils, or epidermal ulceration were observed (Figure 4B). By contrast, group 3 (preventive group) showed near-normal mammary gland architecture (Figure 4C) with decreased lobular alveolar damage when DMBA induction was followed by TQ treatment (50 mg/kg b. wt.). The group 3 rats showed significant reductions in all the tumor types detected in group 2, except that some of the glands showed an increased number of acini, which are normally only seen during pregnancy and lactation.

### 2.4. Effect of TQ on DMBA-Induced Histopathological Changes in Female Rat Kidneys

**Figure 5:**
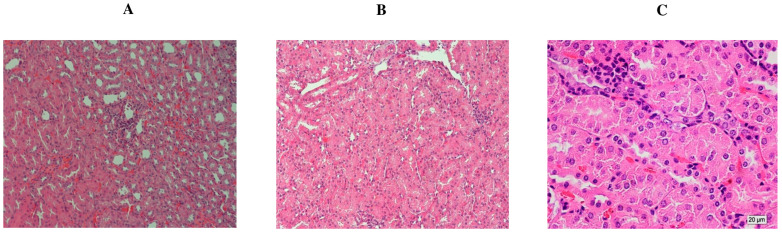
Effect of TQ on histopathology of the kidney tissue from rats with DMBA-induced breast cancer. Histological sections of rat kidney tissues reveal the typical structure of the control group’s kidney gland (×200) (**A**). In (**B**), group 2: (DMBA (65 mg/kg, b. wt.) (×200)): the kidney gland shows hypertrophy with the presence of cancer cells infiltrating and a slight decrease in renal corpuscle size. (**C**) the preventive group consisting of DMBA (65 mg/kg, b. wt.) and TQ (50 mg/kg, b. wt.) (400) displayed some mild deformation in certain renal corpuscles, but the kidney tubules exhibited a healthy lining epithelium.

The kidneys of the control rats showed normal renal corpuscles and glomerular capillaries. The cortical and medullary tubules also showed normal narrow luminas and epithelial linings (Figure 5A). The kidneys of the DMBA-induced rats (Figure 5B) showed mild aggregations of infiltrating viable neoplastic cells, a decrease in renal corpuscle and glomerular size, and dilation of the tubular lumina. By contrast, the kidneys in group 3 (preventive group) showed only a slight deformity of some of the renal corpuscles (Figure 5C) when DMBA induction was followed by TQ treatment (50 mg/kg b. wt.). The kidney tubules showed a healthy epithelial lining like that of the group 1 control animals, with only a slight luminal dilation in the cortical region (Figure 5C). However, the abundance of neoplastic cells detected (Figure 5C) exhibits a notable increase in comparison to the control group.

### 2.5. Effect of TQ on DMBA-Induced Histopathological Changes in Female Rat Livers

**Figure 6:**
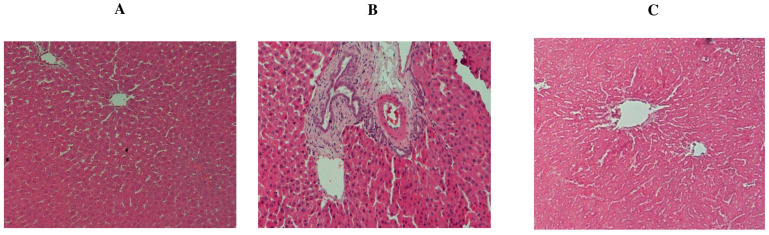
The rat liver tissue segment was stained with hematoxylin and eosin to examine its histological features. In the control group, the hepatic gland’s normal anatomy was revealed through histological sections (×200) (**A**). (**B**) positive control: (DMBA 65 mg/kg, b. wt.) (×200): cytoplasmic degeneration, inflammatory cell aggregation, and some fatty degenerations were seen in the hypertrophied liver gland (×200). (**C**) preventive group: (DMBA (65 mg/kg, b. wt.) and TQ (50 mg/kg, b. wt.) (×400)); liver tissue looks healthy with mild activation of Kupffer cells.

The hepatic organs of control rats showed healthy normal hepatocytes with some bi-nucleated cells indicating regeneration (Figure 6A). The hepatic organs of the DMBA-induced rats (group 2) showed cytoplasmic degeneration and some aggregation of inflammatory cells and fatty degeneration, necrosis, and distortion (Figure 6B). By contrast, group 3 (preventive group) rats induced with DMBA and treated with TQ (50 mg/kg, b. wt.) showed a healthy epithelial lining similar to that of the control group (group 1) but with a mild activation of Kupffer cells (Figure 6C).

### 2.6. TQ Induced DNMT1 Downregulation in BC Cells and DMBA-Treated Female Rats

**Figure 7:**
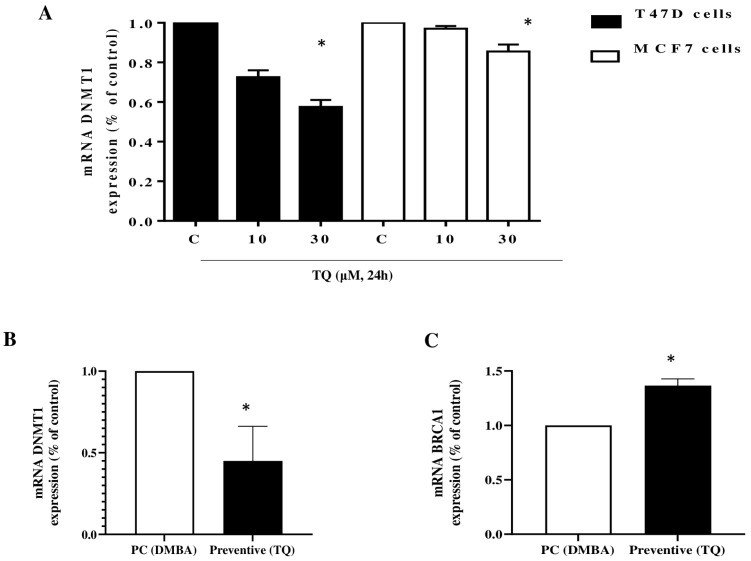
The effect of TQ on the mRNA expression of DNMT1 and *BRCA1*. MCF7 and T47D cells (**A**) were treated with 10 or 30 µM TQ for 24 h. The histograms show the quantification data of mRNA expression of DNMT1 as assessed by real-time RT-PCR. Data are shown as mean ± S.E.M. (n = 3); (* *p* < 0.05) versus respective control. Effect of TQ (50 mg/kg, b. wt.) on DNMT1 (**B**), and *BRCA1* (**C**) mRNA levels in mammary tissue homogenate from rats with DMBA-induced breast cancer. Data shown as means ± S.E.M. (n = 12); * *p* < 0.05, compared to the positive control group.

DNMT1 was reported to be overexpressed in the MCF-7 and T47D breast cancer cell lines [31,32], and its downregulation led to cell proliferation inhibition and the induction of apoptosis [32]. In line with this, our results showed that at 30 µM of TQ, mRNA expression levels of the DNMT1 gene were significantly decreased in both MCF7 and T47D cell lines (Figure 7A). Interestingly, administering DMBA-treated female rats with TQ 50 mg/kg significantly decreased the expression of DNMT1 (Figure 7B) and increased the expression of *BRCA1* in tumorous mammary tissues (Figure 7C).

## 3. Discussion

The silencing of TSG by DNMT1 promotes breast cancer progression and contributes to metastasis [12,33]. Therefore, the decrease in DNMT1 expression in BC is expected to inhibit cell proliferation and metastasis through the reactivation of TSG. In this context, the tumor suppressor gene *BRCA1* has been shown to be increased in response to natural compounds exhibiting anti-cancer activities such as liquiritigenin [34] and genistein [35]. In this study, we showed that the natural compound thymoquinone inhibited cell proliferation and induced apoptosis of MCF7 and T47D breast cancer cells. The TQ-mediated inhibitory effects on MCF7 and T47D cells were associated with the downregulation of DNMT1, indicating that TQ could be a promising candidat for the treatment of breast cancer by inhibiting DNMT1. The in vitro results were substantiated in vivo in female rats, as TQ reduced the number of liver and kidney metastases in DMBA-treated rats, and these effects were associated with a decrease in the expression of DNMT1.

While DMBA treatment led to neoplasia and hyperplasia in the rat breast tissues, subsequent treatment with TQ improved the breast tissue morphology and reduced both the rate of DMBA carcinogenicity and the degree of mammary tumor growth. In line with our findings, a restriction in neoplastic alteration during the steps involved in oncogenesis in DMBA-treated male Syrian hamsters have been shown upon their treatment with TQ [36]. These findings indicate that TQ administration suppresses DMBA-induced mammary carcinogenesis, providing further evidence for the use of phytochemicals, including TQ, in the prevention and the progression of BC.

Interestingly, despite treatment with DMBA, the preventive group treated with TQ showed no malignant tumors and resembled the control group. DMBA caused moderate inflammatory alterations in the tissues but did not induce any epithelial alterations. These changes were particularly noticeable in the kidneys and liver, as histopathological differences were noticed between the DMBA (group 2) and DMBA-TQ (preventive group 3) groups. These results were somewhat surprising, as they indicate that the oxidative damage caused by DMBA is not necessarily linked to the histological findings. A previous study showed that the effects of DMBA were mainly on gut glutathione metabolism and observed no variations in gut pathology between the treated and untreated groups, despite considerable changes in gut glutathione metabolism [37].

In histopathology, the injured liver and kidney cells in the DMBA-treated rats displayed significant cytoplasmic vacuolization, high eosinophilic cytoplasm, necrosis, blood sinusoid compaction, and hyperchromatic nuclei. These changes appeared to follow the same pattern as those previously published for rodent livers and kidneys after the oral administration of DMBA carcinogens [38]. Different-sized vacuoles were found in the cytoplasm of the hepatocytes and kidney cells, which may increase the permeability of cell membranes and may reflect intracytoplasmic lipid accumulation [39,40] and an increase in the number of pleomorphic mitochondria with a dense matrix [41,42]. These types of changes could explain the excessive presence of eosinophilic cytoplasm in these tissues [43].

The DMBA-treated rat tissues typically showed swollen hepatocytes and kidney cells with dilated ER, deformed microvilli, and poor intercellular linkages. These changes could again be related to mitochondrial malfunction, which would cause a decrease in the functioning of the plasma membrane energy-dependent sodium pump, resulting in intracellular sodium buildup and potassium outflow [41,44]. Furthermore, DMBA treatment may promote p53 overexpression [45], which could lead to alterations in mitochondrial architecture [46] and impairment of ER-mitochondria contact sites [41,47].

The cell structure of the DMBA-treated rats showed considerable modifications, including irregular shapes, hyperchromatic nuclei, and an increase in the number of bi-nucleated cells, consistent with previous findings [41]. An increase in mitotic activity could be related to the increased number of bi-nucleated hepatocytes and kidney cells [48]. In addition, the nucleoli were uneven, large, and segregated, in agreement with previous studies [49]. Administration of TQ to DMBA-treated rats restored these alterations to a considerable degree, as evidenced by the histopathology of the tissues from the treated animals. This restoration of normal architecture indicated that TQ is effective against cancer in vivo.

Clinically, patients with BC were reported to overexpress DNMT1 compared to patients with breast fibroadenoma [14], and this overexpression was associated with BC development, while its downregulation inhibits proliferation and invasion of BC [11,12,13]. In the present study, 30 µM of TQ significantly decreased mRNA expression levels of the DNMT1 gene in breast cancer cell lines. DNMT1 overexpression has been correlated with a decrease in the expression of the tumor suppressor gene *BRCA1* in lymph node metastasis [14]. Our results showed that administering TQ to DMBA-treated female rats significantly increased the expression of *BRCA1*, and this effect was associated with a decrease in DNMT1 expression. These changes provide further evidence that DNMT1 is involved in the progression of BC through an epigenetic inactivation of *BRCA1* and that DNMT1 inhibition by TQ could be a key event modulating the inhibition of BC proliferation and metastasis.

## 4. Materials and Methods

### 4.1. Cell Culture and Treatment

MCF-7 and T47D human breast cancer cell lines were purchased from the America Type Culture Collection (Manassas, VA, USA). DMEM (UFC-Biotech, Riyadh, Saudi Arabia) supplemented with 15% (*v*/*v*) foetal calf serum (FCS, Biowhitaker, Lonza, Belgium), 2 mM glutamine, penicillin (100 IU/mL), and streptomycin (Sigma St. Louis, MO, USA) (100 g/mL) was used to maintain the cell lines. Conditions of 37 °C, 5% CO_2,_ and 95% relative humidity were maintained on all cell lines in the incubator. A 10 mM solution of TQ (Sigma-Aldrich, Louis, MO, USA) in 10% Dimethyl sulfoxide (DMSO) (Millipore, Molsheim, France) was used for all treatments and for preparing the requisite working concentrations with cell culture medium. In both controls, the overall DMSO concentration remained less than 0.1%. 7,12-dimethylbenzanthracene (DMBA) was obtained from Sigma-Aldrich (St. Louis, MO, USA). Corn oil was used to prepare the stock solutions of thymoquinone.

### 4.2. Cell Proliferation Assay

To investigate the impact of TQ on the proliferation of ductal carcinoma cells, T47D, and MCF-7cells, a colorimetric cell proliferation assay using the WST-1 Cell Proliferation Reagent kit (Sigma-Aldrich; Merck KGaA, Darmstadt, Germany) was used. In brief, the breast cancer cells were cultured in 96-well plates at a density of 3 × 10^4^ cells/well. The cells were subsequently subjected to increasing concentrations of TQ at 10 and 30 µM for 24 h following 24 h of incubation at 37 °C. Quickly prepared WST-1 reagent was subsequently utilized to assess the rate of cell growth. A total of 10 μL of the WST-1 solution was added after 24 h of incubation, and then the solution was subjected to an extra 3 h of incubation at 37 °C. These results were then analyzed using Gen5 software (BioTek Instruments, Inc., Winooski, VT, USA), and the absorbance was measured at 450 nm using a microplate ELISA reader (ELx800™; BioTek Instruments, Inc.). In this reaction, the tetrazolium salt WST-1 is transformed into formazan product via cellular mitochondrial dehydrogenases. The number of metabolically active, viable cells is directly proportional to the amount of formazan dye found in the media. The percentage of cell viability was determined by assuming the control (untreated) sample viability to be 100%.

### 4.3. Annexin-V-FITC Analysis (Apoptosis Assay)

Cells (2 × 105) were seeded into a 6-well plate and cultured overnight. The cells were then treated with increasing concentrations of TQ at 10 and 30 µM for 24 h. Annexin V Binding Guava Nexin^®^ Assay by capillary cytometry (Guava Easycyte Plus HP system, with absolute cell count and six parameters) was then used to evaluate the cellular apoptosis rate following the manufacturer’s instructions (Guava Technologies Inc, Hayward, CA, USA). Briefly, Nexin^®^ reagent (100 µL) (Millipore^®^, Billerica, MA, USA, catalog no. 4500-0450) was added to each well. The cells were further incubated for 20 min at room temperature in a dark environment. The forward and side scatter were recorded at 10,000 events. Then, a Guava^®^ easyCyte 12HT Benchtop Flow Cytometer (Millipore^®^, Billerica, MA) was used to analyze the percentage of the early and the late apoptotic cells. InCyte™ software (Millipore^®^, Billerica, MA, USA, https://www.thelabworldgroup.com/product/guava-easycyte-ht-cytometer/, accessed on 12 January 2024) was finally used to plot the results.

### 4.4. Reverse Transcription and Real-Time PCR

Cells were treated with increasing concentrations of TQ at 10 and 30 µM for 24 h. The total RNA was isolated and purified from cancer cells using the RNeasy kit (Haven Scientific, Jeddah, Makkah, Saudi Arabia).

### 4.5. Experimental Animals

The 42-day-old female Wistar albino rats, weighing 180–200 g, were obtained from the animal facility at King Fahd Medical and Research Centre in Jeddah, Saudi Arabia followed by acclimatization for one week in the laboratory. The rats were housed in polypropylene cages. Rats were provided a standard food pellet diet and free access to water and were kept at an optimum temperature of 22 ± 1 °C, relative humidity of 60 ± 10%, and in an artificially illuminated (12 h dark/light cycle) room. The use of animals for research was carried out in observance with the Ethics Committee’s approval to the protocol (443-19 of HA-02-J-008), Faculty of Biochemistry, King Abdul-Aziz University, Jeddah Saudi Arabia.

### 4.6. Treatment Regime

The rats (n = 36) were randomly divided into three groups of twelve animal each, as follows:

- Control group: animals were administered corn oil (1 mL/kg) three times a week by oral gavage for 14 weeks [50].
- Positive control: animals were administered a single dose of DMBA (65 mg/kg) dissolved in corn oil by oral gavage.
- Preventive group: animals were administered a single dose of DMBA (65 mg/kg) dissolved in corn oil by oral gavage, and then they were given TQ (50 mg/kg), also dissolved in corn oil, orally three times a week for 14 weeks [51].

Rats were examined weekly for tumors by palpation beginning at 4 weeks after DMBA administration. After receiving full doses for 14 weeks, the rats were euthanized under the effect of the diethyl ether by a cervical dislocation to obtain organ samples from all rats in the three groups. Tumor tissue was weighed and subjected to histopathological analysis.

### 4.7. Histopathological Analysis

Breast tissues were soaked in a formaldehyde solution (10%), dehydrated in ethanol, embedded in paraffin wax, and sectioned with a microtome at 5-µm thickness. The sections were then mounted on slides and stained with eosin and hematoxylin and observed using a trinocular microscope.

### 4.8. Analysis of mRNA Levels

The mRNA levels of DNMT1, *BRCA1*, and 36P4 in breast tissues were determined by quantitative reverse transcription polymerase chain reaction (qRT-PCR). The total RNA was isolated and purified from breast tissues using kits (Superscript III Reverse Transcriptase, Invitrogen, Waltham, MA, USA). The RNA was used to create cDNA libraries (Superscript III Reverse Transcriptase, Invitrogen) by using specific primers. RT-PCR was then carried out by employing SYBR Green qPCR (iQ SUPERMIX, BioRad, Hercules, CA, USA) on an ABI7500 system. The used qPCR conditions were as follows: 95 °C for 30 s, 60 °C for 40 s, and finally 72 °C for 40 s. The results were finally normalized to those obtained with 36P4 mRNA. The CT values of the samples were determined, and relative expression was calculated using the 2^−ΔΔCT^ method. The sequences of the primers used for the PCR amplification were as follows: DNMT1 (sense: 5′-GGCCTTTTCACCTCCATCAA-3′; antisense: 5′-GCACAAACTGACCTGCTTCA-3′); *BRCA1* (sense: 5′-CCGCCTTGCTTTAACTGATGT-3′; antisense: 5′-CACTTTCCTCCTGCAATGCC-3′); 36P4 (sense: 5′-AGTACCTGCTCAGAACACCG-3′ antisense: 5′-GCCATTGTCAAACACCTGCT-3′). Amplicons were size controlled on agarose gel and purity was assessed by analysis of the melting curves at the end of the RT-PCR reaction.

### 4.9. Statistical Analysis

All the data were presented as the mean ± S.E.M of three independent experiments for studies on cell lines with twelve rats per group. Statistical analysis and plotting graphs were performed using GraphPad Prism 8 (GraphPad Software, San Diego, CA, USA). A one-way ANOVA followed by a Tukey’s post hoc test was used to compare the differences among the groups. The statistically significant differences of *p*-values were indicated as * *p* < 0.05, ** *p* < 0.01, *** *p* < 0.001, and **** *p* < 0.0001.

## 5. Conclusions

In conclusion, we propose that TQ is a promising drug for breast cancer through its ability to target DNMT1. Besides its in vitro activity, TQ reduced the number of liver and kidney metastases in vivo in DMBA-treated rats. While DMBA led to neoplasia and hyperplasia in breast tissues, the administration of TQ to rats prevented these histological alterations in breast tissues and reduced DMBA carcinogenicity in rats, and these inhibitory effects were associated with a decrease in DNMT1 expression. Taken together, these results suggest that TQ exerts protective effects against BC risk and reduces the development of metastatic tumors in vivo through a mechanism involving a downregulation of DNMT1, which leads to the inhibition of proliferation and the induction of apoptosis of tumor cells. Further research is needed into the mechanisms by which TQ regulates the expression of DNMT1 in breast cancer cells and the related events.

## Data Availability

All the generated data are included in the manuscript.

## References

[1] Fang L., Liu Q., Cui H., Zheng Y., Wu C. (2022). Bioinformatics Analysis Highlight Differentially Expressed CCNB1 and PLK1 Genes as Potential Anti-Breast Cancer Drug Targets and Prognostic Markers. Genes.

[2] Hobbs E.A., Chen N., Kuriakose A., Bonefas E., Lim B. (2022). Prognostic/predictive markers in systemic therapy resistance and metastasis in breast cancer. Ther. Adv. Med. Oncol..

[3] Torre L.A., Bray F., Siegel R.L., Ferlay J., Lortet-Tieulent J., Jemal A. (2015). Global cancer statistics, 2012. CA Cancer J. Clin..

[4] Arnold M., Morgan E., Rumgay H., Mafra A., Singh D., Laversanne M., Vignat J., Gralow J.R., Cardoso F., Siesling S. (2022). Current and future burden of breast cancer: Global statistics for 2020 and 2040. Breast.

[5] Chen J.-Y., Luo C.-W., Lai Y.-S., Wu C.-C., Hung W.-C. (2017). Lysine demethylase KDM2A inhibits TET2 to promote DNA methylation and silencing of tumor suppressor genes in breast cancer. Oncogenesis.

[6] Sturgeon S.R., Balasubramanian R., Schairer C., Muss H.B., Ziegler R.G., Arcaro K.F. (2012). Detection of promoter methylation of tumor suppressor genes in serum DNA of breast cancer cases and benign breast disease controls. Epigenetics.

[7] Xie Q., Bai Q., Zou L., Zhang Q., Zhou Y., Chang H., Yi L., Zhu J., Mi M. (2014). Genistein inhibits DNA methylation and increases expression of tumor suppressor genes in human breast cancer cells. Genes Chromosom. Cancer.

[8] Birgisdottir V., Stefansson O.A., Bodvarsdottir S.K., Hilmarsdottir H., Jonasson J.G., Eyfjord J.E. (2006). Epigenetic silencing and deletion of the *BRCA1* gene in sporadic breast cancer. Breast Cancer Res..

[9] Esteller M., Silva J.M., Dominguez G., Bonilla F., Matias-Guiu X., Lerma E., Bussaglia E., Prat J., Harkes I.C., Repasky E.A. (2000). Promoter Hypermethylation and *BRCA1* Inactivation in Sporadic Breast and Ovarian Tumors. J. Natl. Cancer Inst..

[10] Rice J.C., Ozcelik H., Maxeiner P., Andrulis I., Futscher B.W. (2000). Methylation of the *BRCA1* promoter is associated with decreased *BRCA1* mRNA levels in clinical breast cancer specimens. Carcinogenesis.

[11] Kastl L., Brown I., Schofield A.C. (2010). Altered DNA methylation is associated with docetaxel resistance in human breast cancer cells. Int. J. Oncol..

[12] Li Z., Li Y., Ren K., Li X., Han X., Wang J. (2017). Long non-coding RNA H19 promotes the proliferation and invasion of breast cancer through upregulating DNMT1 expression by sponging miR-152. J. Biochem. Mol. Toxicol..

[13] Zhang W., Chang Z., Shi K.E., Song L., Cui L.I., Ma Z., Li X., Ma W., Wang L. (2016). The correlation between DNMT1 and ERα ex-pression and the methylation status of ERα, and its clinical significance in breast cancer. Oncol. Lett..

[14] Yu Z., Xiao Q., Zhao L., Ren J., Bai X., Sun M., Wu H., Liu X., Song Z., Yan Y. (2015). DNA methyl-transferase 1/3a overexpression in sporadic breast cancer is associated with reduced expression of estrogen recep-tor-alpha/breast cancer susceptibility gene 1 and poor prognosis. Mol. Carcinog..

[15] Si X., Liu Y., Lv J., Ding H., Zhang X.A., Shao L., Yang N., Cheng H., Sun L., Zhu D. (2016). ERα propelled aberrant global DNA hypermethylation by activating the DNMT1 gene to enhance anticancer drug resistance in human breast cancer cells. Oncotarget.

[16] Wang Q., Li G., Ma X., Liu L., Liu J., Yin Y., Li H., Chen Y., Zhang X., Zhang L. (2023). LncRNA TINCR impairs the efficacy of immunotherapy against breast cancer by recruiting DNMT1 and downregulating MiR-199a-5p via the STAT1–TINCR-USP20-PD-L1 axis. Cell Death Dis..

[17] Laranjeira A.B.A., Hollingshead M.G., Nguyen D., Kinders R.J., Doroshow J.H., Yang S.X. (2023). DNA damage, demethylation and anticancer activity of DNA methyltransferase (DNMT) inhibitors. Sci. Rep..

[18] Parker W.B., Thottassery J.V. (2021). 5-Aza-4’-thio-2’-deoxycytidine, a New Orally Bioavailable Nontoxic “Best-in-Class”: DNA Methyltransferase 1-Depleting Agent in Clinical Development. J. Pharmacol. Exp. Ther..

[19] Zwergel C., Valente S., Mai A. (2016). DNA Methyltransferases Inhibitors from Natural Sources. Curr. Top. Med. Chem..

[20] Achour M., Mousli M., Alhosin M., Ibrahim A., Peluso J., Muller C.D., Schini-Kerth V.B., Hamiche A., Dhe-Paganon S., Bronner C. (2013). Epigallocatechin-3-gallate up-regulates tumor suppressor gene expression via a reactive oxygen species-dependent down-regulation of UHRF1. Biochem. Biophys. Res. Commun..

[21] Liu Z., Xie Z., Jones W., Pavlovicz R.E., Liu S., Yu J., Li P.-K., Lin J., Fuchs J.R., Marcucci G. (2009). Curcumin is a potent DNA hypomethylation agent. Bioorganic Med. Chem. Lett..

[22] Abolfathi S., Zare M. (2023). The evaluation of chitosan hydrogel based curcumin effect on DNMT1, DNMT3A, DNMT3B, MEG3, HOTAIR gene expression in glioblastoma cell line. Mol. Biol. Rep..

[23] Abdullah O., Omran Z., Hosawi S., Hamiche A., Bronner C., Alhosin M. (2021). Thymoquinone Is a Multitarget Single Epidrug That Inhibits the UHRF1 Protein Complex. Genes.

[24] Ibrahim A., Alhosin M., Papin C., Ouararhni K., Omran Z., Zamzami M.A., Al-Malki A.L., Choudhry H., Mély Y., Hamiche A. (2018). Thymoquinone challenges UHRF1 to commit auto-ubiquitination: A key event for apoptosis induction in cancer cells. Oncotarget.

[25] Qadi S.A., Hassan M.A., Sheikh R.A., Baothman O.A., A Zamzami M., Choudhry H., Al-Malki A.L., Albukhari A., Alhosin M. (2019). Thymoquinone-Induced Reactivation of Tumor Suppressor Genes in Cancer Cells Involves Epigenetic Mechanisms. Epigenetics Insights.

[26] Alhosin M., Razvi S.S.I., Sheikh R.A., Khan J.A., Zamzami M.A., Choudhry H. (2020). Thymoquinone and Difluoro-methylornithine (DFMO) Synergistically Induce Apoptosis of Human Acute T Lymphoblastic Leukemia Jurkat Cells Through the Modulation of Epigenetic Pathways. Technol. Cancer Res. Treat..

[27] Alsanosi S., Sheikh R.A., Sonbul S., Altayb H.N., Batubara A.S., Hosawi S., Al-Sakkaf K., Abdullah O., Omran Z., Alhosin M. (2022). The Potential Role of *Nigella sativa* Seed Oil as Epigenetic Therapy of Cancer. Molecules.

[28] Pang J., Shen N., Yan F., Zhao N., Dou L., Wu L.-C., Seiler C.L., Yu L., Yang K., Bachanova V. (2017). Thymoquinone exerts potent growth-suppressive activity on leukemia through DNA hypermethylation reversal in leukemia cells. Oncotarget.

[29] Abusnina A., Alhosin M., Keravis T., Muller C.D., Fuhrmann G., Bronner C., Lugnier C. (2011). Down-regulation of cyclic nu-cleotide phosphodiesterase PDE1A is the key event of p73 and UHRF1 deregulation in thymoquinone-induced acute lym-phoblastic leukemia cell apoptosis. Cell. Signal..

[30] Alhosin M., Abusnina A., Achour M., Sharif T., Muller C., Peluso J., Chataigneau T., Lugnier C., Schini-Kerth V.B., Bronner C. (2009). Induction of apoptosis by thymoquinone in lymphoblastic leukemia Jurkat cells is mediated by a p73-dependent pathway which targets the epigenetic integrator UHRF1. Biochem. Pharmacol..

[31] Li Z., Wang P., Cui W., Yong H., Wang D., Zhao T., Wang W., Shi M., Zheng J., Bai J. (2022). Tumour-associated macrophages enhance breast cancer malignancy via inducing ZEB1-mediated DNMT1 transcriptional activation. Cell Biosci..

[32] Xiang F., Zhu Z., Zhang M., Wang J., Chen Z., Li X., Zhang T., Gu Q., Wu R., Kang X. (2021). 3,3’-Diindolylmethane Enhances Paclitaxel Sensitivity by Suppressing DNMT1-Mediated KLF4 Methylation in Breast Cancer. Front. Oncol..

[33] Zhu X., Lv L., Wang M., Fan C., Lu X., Jin M., Li S., Wang F. (2022). DNMT1 facilitates growth of breast cancer by inducing MEG3 hyper-methylation. Cancer Cell Int..

[34] Liang F., Zhang H., Gao H., Cheng D., Zhang N., Du J., Yue J., Du P., Zhao B., Yin L. (2020). Liquiritigenin decreases tumorigenesis by inhibiting DNMT activity and increasing *BRCA1* transcriptional activity in triple-negative breast cancer. Exp. Biol. Med..

[35] Romagnolo D.F., Donovan M.G., Papoutsis A.J., Doetschman T.C., Selmin O.I. (2017). Genistein Prevents *BRCA1* CpG Methyl-ation and Proliferation in Human Breast Cancer Cells with Activated Aromatic Hydrocarbon Receptor. Curr. Dev. Nutr..

[36] Pu Y., Hu S., Chen Y., Zhang Q., Xia C., Deng H., Wang Y., Hu Q. (2021). Thymoquinone loaded calcium alginate and polyvinyl alcohol carrier inhibits the 7,12-dimethylbenz[a]anthracene-induced hamster oral cancer via the down-regulation of PI3K/AKT/mTOR signaling pathways. Environ. Toxicol..

[37] Cao Y., Wang J., Henry-Tillman R., Klimberg V. (2001). Effect of 7,12-Dimethylbenz[a]anthracene (DMBA) on Gut Glutathione Metabolism. J. Surg. Res..

[38] Sharma V., Paliwal R. (2013). Potential Chemoprevention of 7,12-Dimethylbenz[a]anthracene Induced Renal Carcinogenesis by Moringa oleifera Pods and Its Isolated Saponin. Indian J. Clin. Biochem..

[39] Morito S., Yasui H., Itoh T., Kamoshida S., Ohsaki H. (2023). Malignant mesothelioma cells with characteristic intracytoplasmic vacuolization and lipids. Diagn. Cytopathol..

[40] Zhang L.-C., Liu Y.-N., La X.-Q., Li S.-T., Wen L.-N., Liu T., Li H.-Q., Li A.-P., Wu H., Wu C.-X. (2023). The bound polyphenols of foxtail millet (Setaria italica) inner shell inhibit breast cancer by promoting lipid accumulation-induced autophagic death. Food Chem. Toxicol..

[41] Abdelmeguid N.E., Khalil M.I., Badr N.S., Alkhuriji A.F., El-Gerbed M.S., Sultan A.S. (2021). Ameliorative effects of colostrum against DMBA hepatotoxicity in rats. Saudi J. Biol. Sci..

[42] Rout S.K., Priya V., Setia A., Mehata A.K., Mohan S., Albratty M., Najmi A., Meraya A.M., Makeen H.A., Tambuwala M.M. (2022). Mitochondrial targeting theranostic nanomedicine and molecular biomarkers for efficient cancer diagnosis and therapy. Biomed. Pharmacother..

[43] Fontana R.J., Liou I., Reuben A., Suzuki A., Fiel M.I., Lee W., Navarro V. (2023). AASLD practice guidance on drug, herbal, and dietary supplement-induced liver injury. Hepatology.

[44] Bhatia D., Capili A., Choi M.E. (2020). Mitochondrial dysfunction in kidney injury, inflammation, and disease: Potential ther-apeutic approaches. Kidney Res. Clin. Pract..

[45] Kwon Y.-J., Kwon T.-U., Shin S., Lee B., Lee H., Park H., Kim D., Moon A., Chun Y.-J. (2024). Enhancing the invasive traits of breast cancers by CYP1B1 via regulation of p53 to promote uPAR expression. Biochim. Biophys. Acta Mol. Basis Dis..

[46] Wang H., Yu W., Wang Y., Wu R., Dai Y., Deng Y., Wang S., Yuan J., Tan R. (2023). p53 contributes to cardiovascular diseases via mitochondria dysfunction: A new paradigm. Free. Radic. Biol. Med..

[47] Xiong C., Ling H., Hao Q., Zhou X. (2023). Cuproptosis: p53-regulated metabolic cell death?. Cell Death Differ..

[48] Poojari R., Gupta S., Maru G., Khade B., Bhagwat S. (2010). Chemopreventive and hepatoprotective effects of embelin on N-nitrosodiethylamine and carbon tetrachloride induced preneoplasia and toxicity in rat liver. Asian Pac. J. Cancer Prev. APJCP.

[49] Bharati S., Rishi P., Koul A. (2012). Azadirachta indica exhibits chemopreventive action against hepatic cancer: Studies on as-sociated histopathological and ultrastructural changes. Microsc. Res. Tech..

[50] Karnam K.C., Ellutla M., Bodduluru L.N., Kasala E.R., Uppulapu S.K., Kalyankumarraju M., Lahkar M. (2017). Preventive effect of berberine against DMBA-induced breast cancer in female Sprague Dawley rats. Biomed. Pharmacother..

[51] Linjawi S.A.A., Khalil W.K.B., Hassanane M.M., Ahmed E.S. (2015). Evaluation of the protective effect of Nigella sativa extract and its primary active component thymoquinone against DMBA-induced breast cancer in female rats. Arch. Med. Sci..

